# Hepatitis C — feedback from the Consensus Conference on the Management of Acute Hepatitis C (AHC) in Paris, May 2010

**DOI:** 10.1186/1758-2652-13-S4-O31

**Published:** 2010-11-08

**Authors:** M Vogel

**Affiliations:** 1 Onkologische Ambulanz, Medizinische Klinik und Poliklinik III, Bonn, Germany

## 

Since 2000 several outbreaks of acute HCV infections have been reported among HIV-positive patients. The majority of AHC infections have been observed in Europe and have been almost exclusively limited to men who have sex with men. The epidemic is still ongoing, has been recognized in several large HIV-cohorts and recent outbreaks have also been reported from Australia and the USA. Risk factors for sexual transmission in HIV-infected patients appear to be concurrent sexual transmitted diseases such as syphilis or lymphogranuloma venereum or rough sexual practices, both thought to disrupt mucosal integrity and facilitate permucosal infection. Non-injecting drug use has also been more frequently found among HIV-positive patients with AHC compared to HIV-positive without history of AHC. While tremendous efforts have been undertaken to better understand the epidemic, the natural history and pathogenesis of AHC, and to develop concepts on diagnosis and management of AHC in HIV-infected patients there is still a lack of guidance informing us how to best manage our patients.

As data from clinical trials and cohort studies has become available, evidence-based guidelines are timely to permit the best management of these patients. To address this issue, the European AIDS Treatment Network (NEAT) invited members of the European AIDS Clinical Society (EACS) hepatitis group, the European Association for the Study of the Liver (EASL), the European Study Group on Viral Hepatitis (ESGVH) of the European Society of Clinical Microbiology and Infectious Diseases (ESCMID), the European AIDS Treatment group (EATG) and other experts to attend a consensus conference on acute HCV infection in HIV-infected individuals in Paris, France, on May 21st, 2010. On behalf of the consensus panel this presentation will report on the consensus statements developed.

